
JOURNAL INFORMATION
==============================
NLM Title Abbreviation: Innov Aging
Journal ID: Innov Aging
NLM Journal ID: 101703706
Journal ID: innovateage

Innovation in Aging

EISSN: 2399-5300
Publisher: Oxford University Press

ARTICLE INFORMATION
==============================
PMCID: PMC9770033
DOI: 10.1093/geroni/igac059.693
Article ID: igac059.693
Article version: 1
Subjects: Abstracts, Session 3080 (Symposium), AcademicSubjects/SOC02600

OVERVIEW OF NIA BUDGET

Hodes Richard National Institutes of Health, Bethesda, Maryland, United States

Dr. Hodes will discuss budget and overall research priorities for the National Institute on Aging.

Publication date: 2022 Nov
Electronic publication date: 2022 Dec 20
Volume: 6
Issue: Suppl 1
Issue title: Program Abstracts from The GSA 2022 Annual Scientific Meeting, “Embracing Our Diversity. Enriching Our Discovery. Reimagining Aging.”
First page: 174
Last page: 174
Copyright: © The Author(s) 2022. Published by Oxford University Press on behalf of The Gerontological Society of America.
Copyright year: 2022
License: This is an Open Access article distributed under the terms of the Creative Commons Attribution License (https://creativecommons.org/licenses/by/4.0/), which permits unrestricted reuse, distribution, and reproduction in any medium, provided the original work is properly cited.
License URL: https://creativecommons.org/licenses/by/4.0/

Page count: 1

==============================

### OVERVIEW OF NIA BUDGET

Richard Hodes, *National Institutes of Health, Bethesda, Maryland, United States*

Dr. Hodes will discuss budget and overall research priorities for the National Institute on Aging.
